# Association between Abortion History and Perinatal and Neonatal Outcomes of Singleton Pregnancies after Assisted Reproductive Technology

**DOI:** 10.3390/jcm12010001

**Published:** 2022-12-20

**Authors:** Hanxiang Sun, Xiujuan Su, Yang Liu, Guohua Li, Xiaosong Liu, Qiaoling Du

**Affiliations:** 1 Department of Obstetrics, Shanghai First Maternity and Infant Hospital, School of Medicine, Tongji University, Shanghai 200092, China; 2 Clinical Research Center, Shanghai Key Laboratory of Maternal Fetal Medicine, Shanghai First Maternity and Infant Hospital, School of Medicine, Tongji University, Shanghai 200092, China; 3 Department of Reproductive Immunology, Shanghai First Maternity and Infant Hospital, School of Medicine, Tongji University, Shanghai 200092, China

**Keywords:** spontaneous abortion, induced abortion, assisted reproductive technology, perinatal complication, neonatal outcome

## Abstract

Importance: At present, few people have studied the associations between abortion history before pregnancy on the outcomes of women and their infants after assisted reproductive technology (ART). Objective: To explore the association between the history of abortion and the outcomes of singleton pregnancies after ART. Design: This was a retrospective study in a hospital from 2015 to 2020 in Shanghai, China. Pregnant women with live singleton births through ART were included (*n* = 3043). Abortion was classified into spontaneous abortion (SAB) and induced abortion. We compared the maternal and fetal outcomes of singleton pregnancies obtained through ART with different abortion histories. Logistic regression analysis was used to evaluate the associations between the history of pre-pregnancy abortion with perinatal complications and neonatal outcomes. Results: We observed that compared with those who had no abortion history and obtained singleton pregnancies through ART, women who had an abortion history before pregnancy (including SAB or induced abortion history) and only SAB history were more likely to have gestational diabetes mellitus (GDM), thyroid-related diseases, and placenta-related diseases. After adjusting the potential confounding factors, these differences still existed. The adjusted odds ratios (ORs) and 95% confidence interval (CI) of GDM, thyroid-related diseases, and placenta-related diseases in women with a history of abortion and only a history of SAB were 1.239 (1.030–1.492) and 1.240 (1.010–1.522), 1.589 (1.261–2.002) and 1.724 (1.344–2.213), 1.465 (1.183–1.815) and 1.433 (1.132–1.814), respectively. However, we did not observe the association between the history of induced abortion and GDM and thyroid-related diseases. Conclusions and Relevance: Our research showed that pregnant women with singleton pregnancies through ART who had a history of abortion or only a history of SAB were more likely to have GDM, thyroid-related diseases, and placenta-related diseases. Pregnant women who had both SAB and induced abortion before pregnancy had a higher risk of thyroid-related diseases and placenta-related diseases, while women who had only a history of induced abortion had a higher risk of placenta-related diseases. Further research is needed to explore the biological mechanism of different types of abortion related to subsequent pregnancy.

## 1. Introduction

With the high incidence of infertility, more and more couples need assisted reproductive technology (ART) to get pregnant, such as in vitro fertilization (IVF) and intracytoplasmic sperm injection (ICSI) [1]. The safety of ART has attracted the attention of reproductive doctors all over the world [2]. Many studies have shown that ART increased the incidence of adverse pregnancy outcomes, including premature delivery, hypertensive disorder complicating pregnancy, placenta previa, gestational diabetes mellitus (GDM), and high cesarean section rate [3,4,5,6,7,8]. Moreover, for newborns born through ART, they were more likely to have low birth weight and high infant mortality [9,10]. Multiple pregnancies were one of the important reasons for poor pregnancy outcomes of ART [11].

At present, there are many studies on recurrent abortion and perinatal outcomes [12,13]. A study in Ireland found that recurrent abortion was related to the increased risk of adverse perinatal outcomes, including premature delivery, extremely premature delivery, and perinatal death in subsequent pregnancies [14]. S. Jivraj et al. also believed that the risk of premature delivery, small for gestational age (SGA), and cesarean section in women with a history of recurrent abortion was significantly higher than that of normal pregnant women [15]. However, except for recurrent abortion, there were few studies on the obstetric outcomes of spontaneous abortion (SAB) or induced abortion and subsequent pregnancy. In 2018, a study on abortion history and IVF outcomes in China found that abortion history was related to poor IVF outcomes, including live birth rate and clinical pregnancy rate [16]. Another study found that abortion history had negative effects on the clinical pregnancy rate, early abortion rate, and live birth rate of IVF/ICSI [17]. However, these two studies did not further explore the associations between abortion history and perinatal and neonatal outcomes. The latest study found that the history of spontaneous abortion was related to the increased risk of GDM in later pregnancy [18].

At present, there is no research on the history of spontaneous abortion and induced abortion and the obstetric outcomes of singleton pregnancies through ART. Therefore, we aimed to explore the associations of abortion history with maternal and neonatal outcomes among women who had a history of abortion and obtained singleton pregnancy through ART in a large obstetrics and gynecology hospital in Shanghai, China.

## 2. Materials and Methods

### 2.1. Study Population

**Figure 1:**
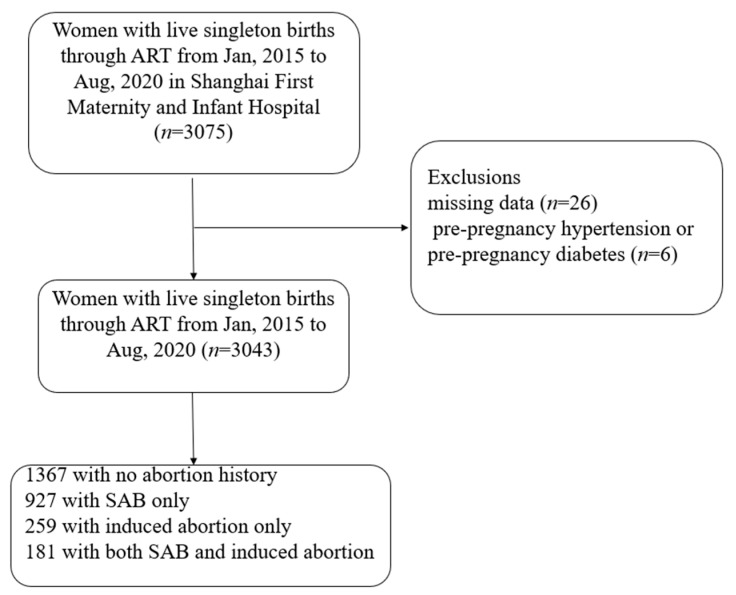
Flow chart of the participant recruitment process (Abbreviation: ART, assisted reproductive technology; SAB, spontaneous abortion).

This was a retrospective study in an obstetrics and gynecology hospital in Shanghai, including the women who obtained single-child live births through ART in this hospital from January 2015 to August 2020 (*n* = 3075). The information about all pregnant women came from the electronic medical record system of Shanghai First Maternity and Infant Hospital. In this study, ART pregnancy refers to pregnancies obtained through IVF or ICSI. Women with missing data (*n* = 26), pre-pregnancy hypertension, or pre-pregnancy diabetes (*n* = 6) were excluded from the study. At last, a total of 3043 women with live singleton births through ART were included for analysis. Among them, 1676 had no history of abortion, 927 had a history of SAB only, 259 had a history of induced abortion only, and 181 had both history of SAB and induced abortion (Figure 1).

### 2.2. Exposure

The primary explanatory variable was the history of abortion, which was classified as no abortion history, SAB history only, induced abortion history only, and both SAB history and induced abortion history.

### 2.3. Outcomes

We were interested in perinatal complications and neonatal outcomes. Perinatal complications included GDM, gestational hypertension, preeclampsia, premature delivery, premature rupture of membranes, polyhydramnios, oligohydramnios, thyroid-related diseases, placenta-related diseases, intrahepatic cholestasis of pregnancy, vaginal group B streptococcus positive, postpartum hemorrhage, and umbilical cord abnormalities. Thyroid-related diseases included hypothyroidism, hyperthyroidism, subclinical hypothyroidism, and other thyroid abnormalities. Diagnosis of thyroid-related diseases included pre-pregnancy diagnosis and pregnancy diagnosis. Placenta-related diseases included placenta previa, placental abruption, placental adhesion, sail placenta, racket placenta, placenta accreta, and other placenta abnormalities. Abnormal umbilical cord included umbilical cord edema, umbilical cord torsion, umbilical cord true knot, umbilical cord spiral, umbilical cord too short, umbilical cord entanglement, and umbilical cord cyst. Neonatal outcomes included fetal distress, low birth weight, very low birth weight, sex of newborn, and 1-min Apgar score.

### 2.4. Statistical Analysis

The basic characteristics of the research population were statistically analyzed by descriptive analysis. Count (%) was used for categorical variables. Cross-table was used to test whether women with an abortion history were related to perinatal complications and adverse neonatal outcomes compared with those without an abortion history. *p* < 0.05 was considered statistically significant. Logistic regression analysis was used to estimate the associations between different classifications of abortions and adverse perinatal and neonatal outcomes. Possible confounding factors included maternal age (≤24, 24–29, 30–34, older than 35 years), pre-pregnancy Body Mass Index (BMI) (<18.5 kg/m^2^,18.5–24.9 kg/m^2^, more than 25 kg/m^2^), delivery mode (vaginal delivery or cesarean section), and parity (primipara or multipara). All analyses were processed by the SPSS26.0 software package (SPSS Inc, Chicago, IL, USA).

## 3. Results

Among the 3043 included pregnant women conceived through ART, 1676 (55.08%) had no history of abortion, 927 (30.46%) had a history of SAB only, 259 (8.51%) had a history of induced abortion only, and 181 (5.95) had both history of SAB and history of induced abortion. Compared with the pregnant women who obtained singleton pregnancies through ART and had no history of abortion, the pregnant women with a history of abortion had a higher probability of age ≥35 (41.48% vs. 32.16%), a higher probability of multipara (10.02% vs. 4.06%), and a higher probability of obesity (17.41% vs. 15.33%). The detailed basic characteristics of the study population are shown in Table 1.

We studied the associations between different types of abortion and perinatal complications and found that compared with women who had singleton pregnancies through ART without abortion history, women with abortion history (including SAB or induced abortion history) and women with SAB history only had a higher probability of GDM, thyroid-related diseases during pregnancy, and placenta-related diseases, with a statistical difference (*p* < 0.05). The probability of GDM (23.94% vs. 17.72%) was higher in women with a history of induced abortion compared with those with no abortion history. The probability of oligohydramnios (5.52% vs. 2.33%), thyroid-related diseases (14.36% vs. 8.95%), and placenta-related diseases (18.23% vs. 11.40%) was higher in women with both SAB and induced abortion history compared with women with no abortion history. However, there was no statistical difference in other pregnancy complications such as premature delivery, premature rupture of membranes, intrahepatic cholestasis of pregnancy, etc. The details are shown in Table 2. However, when comparing the associations between different types of abortions and neonatal outcomes, we did not find differences in neonatal outcomes (Table 3).

Logistic regression analysis was used to evaluate the association between pre-pregnancy abortion history and GDM, thyroid-related diseases, and placental-related diseases. The results showed that after adjusting for potential confounding factors (including maternal age, pre-pregnancy BMI, parity, and mode of delivery), women with a history of SAB only had a higher risk of GDM, thyroid-related diseases, and placenta-related diseases, with adjusted OR and 95% CI of 1.240 (1.010–1.522), 1.724 (1.344–2.213), and 1.433 (1.132–1.814), respectively. For women with a history of abortion (including SAB history or induced abortion history), they were also at greater risk of GDM, thyroid-related diseases, and placenta-related diseases. For those with a history of induced abortion only, only the risk of placenta-related diseases increased after adjusting for confounding factors, with adjusted OR and 95% CI of 1.526 (1.038–2.242). For pregnant women with a history of both SAB and induced abortion, the risk of thyroid-related diseases and placenta-related diseases increased after adjusting confounding factors, and the adjusted OR and 95% CI were 1.657 (1.042–2.634) and 1.747 (1.142–2.673), respectively. The details are shown in Table 4.

## 4. Discussion

As far as we know, this was the first study on the association between the history of abortion and the outcomes of pregnant women and their infants through ART. After analyzing 3043 women who obtained singleton pregnancies through ART, we found that women who had a history of SAB only before pregnancy were at greater risk of GDM, thyroid-related diseases, and placenta-related diseases in subsequent pregnancies. Women with a history of induced abortion only had a greater risk of placenta-related diseases in subsequent pregnancies, while women with both a history of SAB and a history of induced abortion had a greater risk of thyroid-related diseases and placenta-related diseases during pregnancy.

Since Louise Brown was born in 1978, ART has become an effective method for the treatment of infertility. Abortion, especially recurrent abortion, is one of the important causes of infertility [19]. At present, there are many studies on the associations between recurrent abortion and obstetrical outcomes [20,21]. A retrospective Swedish study found that women with recurrent abortion had an increased risk of preeclampsia, stillbirth, SGA, premature delivery, and placental abruption in a subsequent pregnancy [22]. Other studies also believed that women with recurrent abortions had an increased risk of pregnancy complications related to placental dysfunction [23,24]. However, at present, few people have studied the associations between abortion history before pregnancy on the outcomes of women and their infants after ART. A recent study found that the history of SAB was related to the increased risk of GDM in a subsequent pregnancy [18], which was consistent with our research results. At the same time, our study also found that placenta-related diseases were more likely to occur after singleton pregnancies were obtained through ART, regardless of the history of SAB or induced abortion, which was consistent with other studies [25]. A Swedish study on the association between previous abortions and the risk of placental dysfunction showed that two or more abortions were associated with an increased risk of placental dysfunction [23]. Other studies have also suggested that placenta previa, residual placenta, and other placenta-related diseases were associated with abortion [26,27,28,29]. Finally, our study also found that compared with women with no history of abortion, thyroid-related diseases were at greater risk of having only a history of SAB and both a history of SAB and induced abortion. Ines Bucci et al. believed that adequate thyroid hormone was essential for normal menstrual function and fertility, and successful pregnancy, and the association between reproductive failure and thyroid diseases, was particularly relevant [30]. R Mazzilli et al. believed that thyroid function should be carefully monitored for both men and women, whether it was natural pregnancy or pregnancy through ART [31]. Many studies have suggested that abortion is associated with various thyroid diseases [32,33,34]. All these were consistent with our research results.

Our research is the first to explore the association between abortion history and the outcomes of women and their infants after pregnancy through ART. However, our research has some limitations. First of all, this was a retrospective study. All the data came from the hospital’s case system, and the abortion information was self-reported by pregnant women, which may be inaccurate. Secondly, with regard to the diagnosis of thyroid diseases and placenta-related diseases during pregnancy, we did not further classify them to study the association between thyroid diseases and placenta-related diseases during pregnancy and abortion. Thirdly, we did not classify ART to study the impact of different ART types on the results. Fourthly, because the case system did not record the gestational age at the time of SAB or induced abortion, we did not study the influence of the gestational age at the time of abortion on the subsequent pregnancy outcomes.

## 5. Conclusions

Our results showed that women with a history of abortion had an increased risk of perinatal complications after obtaining singleton pregnancies through ART. Among them, women with a history of SAB only had the greatest risk of adverse outcomes, including GDM, thyroid-related diseases, and placenta-related diseases. Therefore, it is necessary to strengthen prenatal care for women who need to become pregnant through ART and have a history of abortion because they are at greater risk of adverse obstetric complications.

## Figures and Tables

**Table 1:** Basic characteristics of the study population classified according to different abortion histories.

	History of Abortion (*n* = 1367)	History of Abortion			No Abortion History (*n* = 1676)
		SAB Only (*n* = 927)	Induced Abortion Only (*n* = 259)	Both SAB and Induced Abortion (*n* = 181)	
Age, y, *n* (%)					
≤24	4 (0.29)	4 (0.43)	0 (0)	0 (0)	15 (0.89)
25–29	160 (11.70)	114 (12.30)	28 (10.81)	18 (9.94)	273 (16.29)
30–34	636 (46.53)	426 (45.95)	119 (45.95)	91 (50.28)	849 (50.66)
≥35	567 (41.48)	383 (41.32)	112 (43.24)	72 (39.78)	539 (32.16)
Pre-pregnancy BMI, kg/m^2^, *n* (%)					
<18.5	116 (8.49)	81 (8.74)	23 (8.88)	12 (6.63)	151 (9.01)
18.5–24.9	1013 (74.10)	679 (73.25)	200 (77.22)	134 (74.03)	1268 (75.66)
≥25	238 (17.41)	167 (18.02)	36 (13.90)	35 (19.34)	257 (15.33)
Parity, *n* (%)					
Nulliparous	1230 (89.98)	869 (93.74)	217 (83.78)	144 (79.56)	1608 (95.94)
Multiparous	137 (10.02)	58 (6.26)	42 (16.22)	37 (20.44)	68 (4.06)
Mode of delivery, *n* (%)					
Vaginal delivery	589 (43.09)	390 (42.07)	125 (48.26)	74 (40.88)	793 (47.32)
Cesarean section	778 (56.91)	537 (57.93)	134 (51.74)	107 (59.12)	883 (52.68)
Year of delivery, *n* (%)					
2015	130 (9.51)	86 (9.28)	26 (10.04)	18 (9.94)	158 (9.43)
2016	228 (16.68)	155 (16.72)	47 (18.15)	26 (14.36)	260 (15.51)
2017	235 (17.19)	170 (18.34)	46 (17.76)	19 (10.50)	289 (17.24)
2018	235 (17.19)	149 (16.07)	48 (18.53)	38 (20.99)	277 (16.53)
2019	308 (22.53)	208 (22.44)	58 (22.39)	42 (23.20)	386 (23.03)
2020	231 (16.90)	159 (17.15)	34 (13.13)	38 (20.99)	306 (18.26)

**Table 2:** Associations between the different classifications of abortions and perinatal complications of women conceived through ART.

	History of Abortion (*n* = 1367)	History of Abortion			No Abortion History (*n* = 1676)
		SAB Only (*n* = 927)	Induced Abortion Only (*n* = 259)	Both SAB and Induced Abortion (*n* = 181)	
Gestational diabetes mellitus	306 (22.38) *	206 (22.22) *	62 (23.94) *	38 (20.99)	297 (17.72)
Gestational hypertension and preeclampsia	142 (10.39)	101 (10.90)	20 (7.72)	21 (11.60)	157 (9.37)
Premature birth	73 (5.34)	53 (5.72)	14 (5.41)	6 (3.31)	79 (4.71)
Premature rupture of membranes	184 (13.46)	122 (13.16)	37 (14.29)	25 (13.81)	245 (14.62)
Polyhydramnios	17 (1.24)	11 (1.19)	3 (1.16)	3 (1.66)	20 (1.19)
Oligohydramnios	33 (2.41)	20 (2.16)	3 (1.16)	10 (5.52) *	39 (2.33)
Thyroid disease during pregnancy	188 (13.75) *	137 (14.78) *	25 (9.65)	26 (14.36) *	150 (8.95)
Placental related diseases	224 (16.39) *	151 (16.29) *	40 (15.44)	33 (18.23) *	191 (11.40)
Intrahepatic cholestasis of pregnancy	15 (1.10)	9 (0.97)	3 (1.16)	3 (1.66)	20 (1.19)
Group B Streptococcus infection	16 (1.17)	13 (1.40)	2 (0.77)	1 (0.55)	17 (1.01)
Postpartum hemorrhage	7 (0.51)	6 (0.65)	1 (0.39)	0 (0)	7 (0.42)
Umbilical cord-related abnormality	25 (1.83)	23 (2.37)	2 (0.77)	1 (0.55)	33 (1.97)

* *p* <0.05.

**Table 3:** Associations between the different classifications of abortions and outcomes of singleton newborns through ART.

	History of Sbortion (*n* = 1367)	History of Abortion			No Abortion History (*n* = 1676)
		SAB Only (*n* = 927)	Induced Abortion Only (*n* = 259)	Both SAB and Induced Abortion (*n* = 181)	
Fetal distress	66 (4.83)	49 (5.29)	8 (3.09)	9 (4.97)	99 (5.91)
Low birth weight	68 (4.97)	49 (5.29)	11 (4.25)	8 (4.42)	68 (4.06)
Very low birth weight	11 (0.80)	5 (0.54)	3 (1.16)	3 (1.66)	10 (0.60)
Gender of newborn (female babies)	651 (47.62)	457 (49.30)	114 (44.02)	80 (44.20)	775 (46.24)
Apgar score at one minute <7	7 (0.51)	4 (0.43)	1 (0.39)	2 (1.10)	11 (0.66)

**Table 4:** Crude and adjusted ^a^ OR (95% CI) for the associations between the classification of abortions and unfavorable outcomes.

	History of Abortion (*n* = 1367)	History of Abortion			No Abortion History (*n* = 1676)
		SAB Only (*n* = 927)	Induced Abortion Only (*n* = 259)	Both SAB and Induced Abortion (*n* = 181)	
Gestational diabetes mellitus Crude OR (95% CI)	1.339 (1.120–1.601) *	1.327 (1.087–1.619) *	1.461 (1.070–1.996) *	1.234 (0.845–1.802)	1 (Reference)
Adjusted OR (95% CI)	1.239 (1.030–1.492) *	1.240 (1.010–1.522) *	1.316 (0.948–1.827)	1.052 (0.705–1.569)	1 (Reference)
Thyroid disease during pregnancy Crude OR (95% CI)	1.622 (1.292–2.037) *	1.764 (1.378–2.259) *	1.087 (0.696–1.696)	1.706 (1.090–2.671) *	1 (Reference)
Adjusted OR (95% CI)	1.589 (1.261–2.002) *	1.724 (1.344–2.213) *	1.075 (0.682–1.696)	1.657 (1.042–2.634) *	1 (Reference)
Placental-related diseases Crude OR (95% CI)	1.524 (1.238–1.876) *	1.513 (1.202–1.905) *	1.420 (0.982–2.054)	1.734 (1.155–2.602) *	1 (Reference)
Adjusted OR (95% CI)	1.465 (1.183–1.815) *	1.433 (1.132–1.814) *	1.526 (1.038–2.242) *	1.747 (1.142–2.673) *	1 (Reference)

^a^ Adjusted for maternal age, pre-pregnancy body mass index, parity (nulliparous, multiparous), and mode of delivery (vaginal delivery, cesarean section). * *p* < 0.05.

## Data Availability

The datasets used and analyzed during the current study are available from the corresponding author upon reasonable request.
